# Insights Into Mechanisms of Oriented Division From Studies in 3D Cellular Models

**DOI:** 10.3389/fcell.2022.847801

**Published:** 2022-03-09

**Authors:** Federico Donà, Susanna Eli, Marina Mapelli

**Affiliations:** IEO, European Institute of Oncology IRCCS, Milan, Italy

**Keywords:** mitotic spindle orientation, epithelial polarity, cysts, organoids, planar divisions

## Abstract

In multicellular organisms, epithelial cells are key elements of tissue organization. In developing tissues, cellular proliferation and differentiation are under the tight regulation of morphogenetic programs, that ensure the correct organ formation and functioning. In these processes, mitotic rates and division orientation are crucial in regulating the velocity and the timing of the forming tissue. Division orientation, specified by mitotic spindle placement with respect to epithelial apico-basal polarity, controls not only the partitioning of cellular components but also the positioning of the daughter cells within the tissue, and hence the contacts that daughter cells retain with the surrounding microenvironment. Daughter cells positioning is important to determine signal sensing and fate, and therefore the final function of the developing organ. In this review, we will discuss recent discoveries regarding the mechanistics of planar divisions in mammalian epithelial cells, summarizing technologies and model systems used to study oriented cell divisions *in vitro* such as three-dimensional cysts of immortalized cells and intestinal organoids. We also highlight how misorientation is corrected *in vivo* and *in vitro*, and how it might contribute to the onset of pathological conditions.

## Introduction

The mitotic spindle is a bipolar structure formed by microtubules (MTs) that in mitosis captures the duplicated chromosomes and segregates them equally between daughter cells. In unicellular and multicellular organisms the mitotic spindle can be regarded as a key player for the successful outcome of cell division (Pietro et al., 2016). In stem cells and progenitors, the mitotic spindle orientation contributes to define the fate choice of daughter cells and their positioning within the tissue, resulting in either symmetric or asymmetric division (Morin and Bellaïche, 2011). Oriented divisions have been extensively studied in invertebrate systems (Gönczy, 2008; Knoblich, 2008; Knoblich, 2010; Morin and Bellaïche, 2011; Pietro et al., 2016), however mechanistic insights into orientation mechanisms in vertebrates are still limited. Spindle positioning is known to impact on cell proliferation, cell fate and tissue development although a comprehensive understanding of the molecular details underlying these processes is just building (Pietro et al., 2016; Lechler and Mapelli, 2021). Timing and execution of spindle placement rely on intrinsic and extrinsic signals sensed by the dividing cell (Pietro et al., 2016).

In the epithelial tissues, contacts between the dividing cell and the adjacent ones are important factors determining the division orientation (Osswald and Morais-de-Sá, 2019). In polarized epithelial monolayers, cells divide by planar divisions with the mitotic spindle parallel to the epithelium, and the two daughter cells remain within the same monolayer, leading to tissue growth and expansion (Nakajima, 2018). Studies on division orientation in 3D culture, including organoids derived from various tissues, are just starting to reveal interesting differences between orientation mechanisms and misorientation correction compared to what observed in 2D and in invertebrate systems. In this review, we summarize what is known about mitotic spindle dynamics and oriented cell divisions in vertebrate 3D cysts and organoids. In the first section, we will present an overview of spindle orientation effectors. Then, we will describe mechanisms of oriented cell divisions in cysts grown from mammalian cell lines, while in the end of the review we will focus on more complex 3D cellular structures such as organoids. Finally, the potential role of mitotic spindle proteins in disease associated with defective epithelial morphogenesis and homeostasis will be discussed, with a few examples from *Drosophila* studies.

### Mitotic Spindle Machinery: The Importance of the Gαi/LGN/NuMA Complex

Division orientation depends on mitotic spindle positioning, that is generally attained in metaphase and sometime corrected in telophase (Morin and Bellaïche, 2011; Lough et al., 2019). In many epithelial systems, division orientation follows the Hertwig’s rule, according to which the spindle aligns along the long axis of the dividing cell (Hertwig, 1884). To which extent spindle alignment to the long cell axis is guided by mechano-sensing pathways responding to compressional cues exerted by neighbouring cells, or it is contributed by cytoskeletal forces exerted by MT motors is still debated. Elegant studies in MDCK (Madin–Darby Canine Kidney) Extra-Cellular-Matrix-free (ECM-free) monolayers “in suspension” showed that the division orientation occurs along the longest cell axis and is instructed by the interphase geometry (Wyatt et al., 2015). In these cells, components of the force generators complexes including NuMA and Gαi accumulates at cortical polar sites. Consistently, studies in *Xenopus* epithelia indicate that cells divide according to interphase cellular shape that is defined by three-cell junction distribution, where LGN and E-cadherin accumulates (Nestor-Bergmann et al., 2019). Collectively, this evidence suggests that in mammalian epithelial cells interphase shape drives force generators distributions to orchestrate divisions along the longest cell axis. Notably, these findings in vertebrate cells are consistent with previous observations in *Drosophila* tissues (Bosveld et al., 2016), although do not seem to apply to the development of *Drosophila* follicular epithelium at early-stage egg chambers (Finegan et al., 2019).

Several studies elucidated the molecular mechanisms of orientation, in which a fundamental role is played by Gαi/LGN/NuMA proteins, an evolutionarily conserved ternary complex. Gαi is the subunit of heterotrimeric G-proteins that localizes at the plasma membrane, LGN acts as a molecular scaffold, and NuMA is the mitotic dynein-adaptor involved in MT-pulling force onset. The majority of studies addressing the mechanistics of orientation were conducted in adherent cells in isolation, such as HeLa cells (Du et al., 2001; Du and Macara, 2004; Zheng et al., 2010; Kiyomitsu and Cheeseman, 2012; Kotak et al., 2012; Gallini et al., 2016; Pirovano et al., 2019; Takayanagi et al., 2019), or in a monolayers of MDCK cells, in which the spindle axis aligns parallel to the substratum in an integrin-dependent manner (Reinsch and Karsenti, 1994; Tuncay et al., 2015; Chishiki et al., 2017; Lázaro-Diéguez and Müsch, 2017).

**FIGURE 1:**
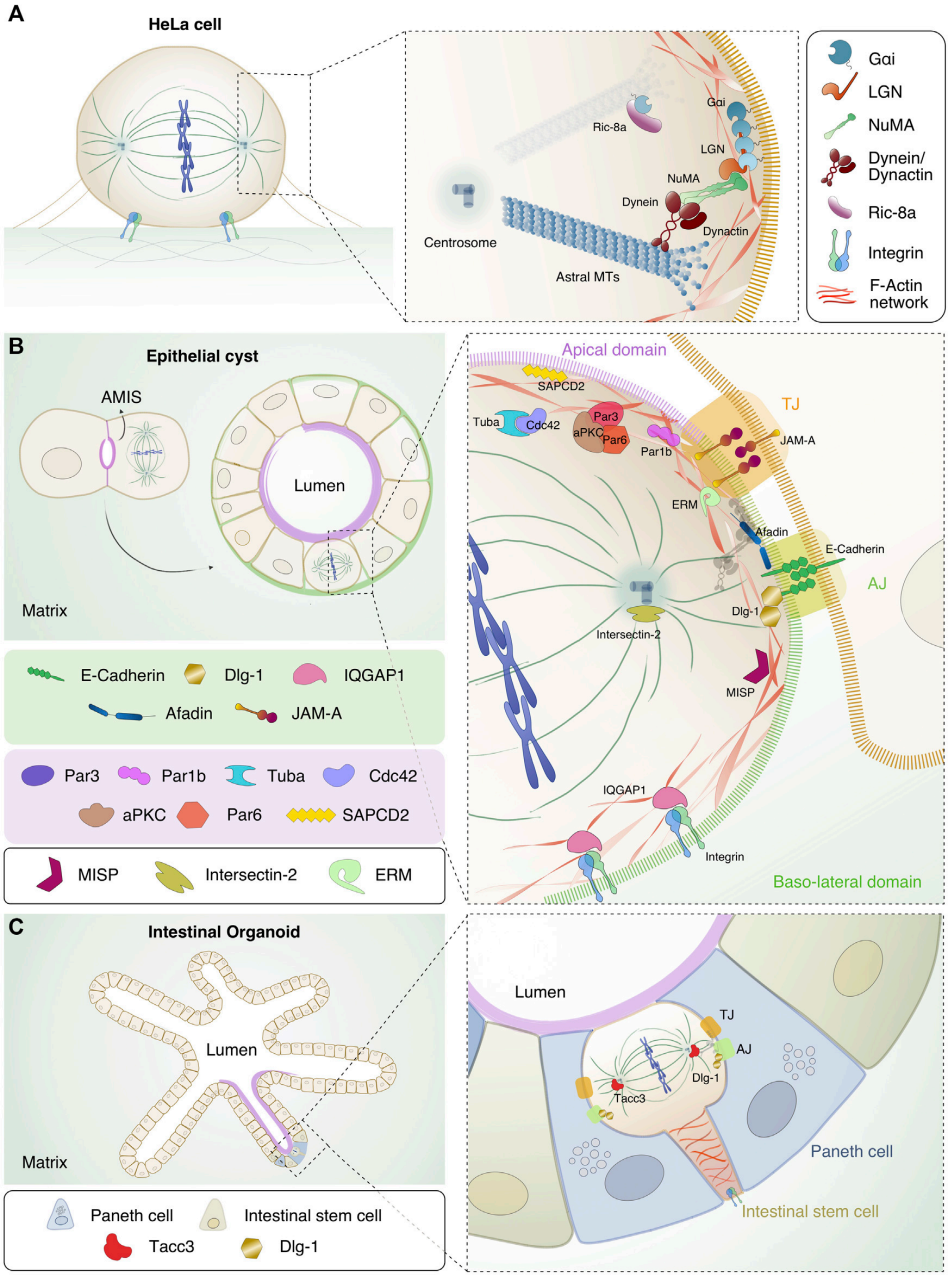
*Localization and interaction of the spindle orientation and polarity proteins in different model systems.* **(A)** HeLa cell in metaphase. Chromosomes (in blue) are aligned at the metaphase plate in the centre of the cell, MTs (in dark green) form the mitotic spindle and integrins important for adhesion of the mitotic cell to the substratum, are shown in light blue and green. In the inset, the details of the interaction interfaces between orientation proteins Gαi/LGN/NuMA, dynein/dynactin (in bordeaux) and astral MTs are shown. The Gαi/NuMA/LGN complex is recruited at the lateral sides above spindle poles. NuMA is in green, LGN in orange and Gαi in petrol blue. Ric8-A (in purple) is shown in the cytoplasm, close to the plasma membrane-bound Gαi. **(B)** Evolution from two-cell stage, in which the mitotic spindle orients parallel to the AMIS, to mature cyst with a single lumen. The diving cell in the mature cyst has the mitotic spindle parallel to the apical side. In the scheme, the apical domain is highlighted in purple while the basolateral side in green. In the close-up, the mitotic spindle proteins displayed in A are shown in relation with the polarity or junctional protein discussed in the text. At the level of cell-cell junctions, the tight junction (TJ, orange box) and the adherens junction (AJ, bright green box) are shown with key components highlighted. At the TJ, JAM-A (in rainbow orange), the polarity complex with Par3 (dark purple), Par6 (light red), aPKC (brown), Cdc42 (lilac) and Tuba (cyan) are depicted. Par1b (fuchsia) and SAPCD2 (yellow) are pictured at the apical side. At the AJ levels, E-cadherin (in green), Afadin (in blue) and Dlg-1 (in gold) are shown. At the basal side of the cell, IQGAP1 (in pink) and integrins are depicted. Intersectin-2 (in olive green) is present at the centrosomes. F-actin is shown in red, and the interacting proteins MISP (in purple wine) and ERM (in aqua green) connecting the mitotic cortex to the plasma membrane are indicated. **(C)** Left: intestinal organoids showing the crypt-villi structure that recapitulates the intestine architecture. The apical side of the organoids is shown in purple, the intestinal stem cells (in ocre) and the Paneth cells (in blue) are highlighted. In the inset on the right, mitotic ISC located apically in the monolayer is shown with the actin cable connecting the dividing cells to the basal membrane. Dlg-1 and Tacc3 (in tomato) are shown.

In metaphase, the Gαi/LGN/NuMA complex localizes at the plasma membrane above the spindle poles (Du et al., 2001; Du and Macara, 2004; Kotak et al., 2012; Gallini et al., 2016; Pirovano et al., 2019; Zheng et al., 2010; Machicoane et al., 2014) and recruits the MT motor dynein/dynactin (Kotak et al., 2012; Okumura et al., 2018; Woodard et al., 2010) (Figure 1A). Exploiting the minus-end directed movement of dynein, cortically localized dynein motors generate pulling forces on astral MTs branching from the spindle poles that in metaphase contribute to spindle placement (Théry et al., 2007; Kiyomitsu and Cheeseman, 2012; Kotak et al., 2012). Notably, ectopic recruitment of NuMA to the cell cortex by optogenetic techniques is necessary and sufficient to orient the spindle, while cortical targeting of dynein is not sufficient to generate enough pulling forces to place the spindle (Fielmich et al., 2018; Okumura et al., 2018), implying that the activity of MT motors requires a defined spatial cortical organization. In line with these findings, recent studies revealed that not only the levels of NuMA/dynein/dynactin motors present at the cortex, but also their spatial distribution plays a role in the onset of effective MT-pulling forces (Pirovano et al., 2019; Renna et al., 2020).

In mitosis Gαi proteins are uniformly enriched at the cell cortex, but only a GDP-loaded pool of Gαi (Gαi^GDP^) accumulates above the spindle poles and is the one that selectively binds to LGN (Du et al., 2001; Willard et al., 2004; Mochizuki et al., 1996). The recruitment of LGN at the cortex by Gαi is controlled by GAPs (GTPase activating proteins) and GEFs (Guanine Exchange Factors) that tune the GTP-state of Gαi. An important Gαi GEF implicated in spindle placement is Ric-8A, which appears to play a key role in targeting LGN to the cortex (Chishiki et al., 2017; Woodard et al., 2010). In metaphase, LGN is spatially restricted to the cortical side facing the spindle poles by direct binding to Gαi (Zheng et al., 2010) (Figure 1A). Lateral recruitment of LGN and in turn NuMA/dynein motors promotes planar spindle orientation (Zheng et al., 2010). Notably, the conformation of LGN depends on its binding partners (Du and Macara, 2004; Pan et al., 2013): in the unliganded form LGN is kept in an inhibited conformation by intra-molecular interactions between the N-terminal TPR domain and the C-terminal GoLoco region. Cooperative binding of the four GoLoco motifs to cortical Gαi^GDP^ molecules recruits LGN to the cortex and induces a conformational change releasing the TPR domain that in turn associates with NuMA. These events result in the enrichment for NuMA/dynein/dynactin motors to specific cortical sites and onset of astral MT-pulling forces (Du and Macara, 2004; Pan et al., 2013) (Figure 1A). Notably, the TPR domain of LGN interacts not only with NuMA but also with Afadin and E-cadherin in a mutually exclusive manner, with functional implications that will be discussed below. NuMA shares the domain structures with other dynein-activator proteins (Kiyomitsu and Boerner, 2021), including a hook domain and a CC1-like box motifs, both at the N-terminus, responsible for the binding to dynein and dynactin. After a central 1500-residue long coiled-coil, NuMA codes for a C-terminal region binding to LGN, microtubules, as well as to the plasma membrane in anaphase (Kotak et al., 2013; Seldin et al., 2013; Carminati et al., 2016; Seldin et al., 2016; Pirovano et al., 2019; Renna et al., 2020). All these diverse functionalities of NuMA C-terminus contribute to spindle placement during mitotic progression, partly modulated by mitotic kinases’ phosphorylation (Lechler and Mapelli, 2021).

### Polarity and Epithelial Junctions in Spindle Orientation in Polarized Monolayers and Cysts

In 2D systems such as polarized MDCK cells grown in monolayer, cells divide with the spindle axis aligned to the substratum by planar symmetric divisions that generate two daughter cells remaining in the same monolayer (Reinsch and Karsenti, 1994; Tuncay et al., 2015; Lázaro-Diéguez and Müsch, 2017). In this setting, spindle alignment is maintained by astral MTs captured by cortical cues localized at the lateral domains of the dividing cell, including cell-cell adhesion molecules (Gloerich et al., 2017; Lázaro-Diéguez and Müsch, 2017). Additional information has been obtained in more physiologically relevant 3D models, such as cysts.

The most common cells used to study oriented divisions in cysts are MDCK and Caco-2 (human colon adenocarcinoma) cells that, when plated on a substrate that mimics the ECM such as matrigel, grow as monolayered spheres by planar divisions occurring with the spindle axis perpendicular to the apico-basal polarity (Zegers et al., 2003; Jaffe et al., 2008). A cyst is characterized by a central lumen and a surrounding monolayer of polarized cells (Zegers et al., 2003) (Figure 1B). Notably, lumen formation in MDCK- or Caco-2-derived cysts relies on spindle orientation, as opposed to cysts obtained from MCF10A cells (human breast immortalized cells) where lumen forms by anoikis, i. e apoptosis of inner cells after a full sphere is formed (Debnath et al., 2002). After the first division, MDCK single cells have been shown to form an apical membrane initiation site (AMIS) between the two daughter cells, in the position where the midbody was located (Overeem et al., 2015), that will later become the lumen of the nascent cyst (Rodriguez-Boulan and Macara, 2014) (Figure 1B left). Cells composing the mature cyst have two types of domains: the apical side facing the central lumen where the PAR (partitioning defective) family proteins localize, and the baso-lateral domain where adhesion proteins such as integrins are in contact with the ECM, and where adjacent cells are in contact with each other by adherens (AJ) and tight junctions (TJ) (McCaffrey and Macara, 2011) (Figure 1B right). Importantly, each of these membrane domains is key for the localization of spindle orientation proteins instructing planar divisions including Gαi, NuMA and LGN (see below) (Overeem et al., 2015; Nakajima, 2018). In Table 1 we summarized the proteins involved in spindle orientation with their function, localization and defects occurring upon depletion.

**TABLE 1:** Proteins involved in division orientation, and model systems in which they were studied (fly and worm orthologues are reported, when present).

Protein	Cellular system	Function	Mitotic localization	Defects upon ablation	REFs
NuMA *dm*Mud, *ce*LIN-5	HeLa, Caco-2/MDCK cyst	Dynein adaptor	Spindle poles Polar, cortex Centrosomes	Misorientation Multilumen	(Du et al., 2001; Du and Macara, 2004; Woodard et al., 2010; Kotak et al., 2012; Kotak et al., 2013; Seldin et al., 2013; Bañón-Rodríguez et al., 2014; Carminati et al., 2016; Gallini et al., 2016; Seldin et al., 2016; Kschonsak and Hoffmann, 2018; Okumura et al., 2018; Pirovano et al., 2019; Renna et al., 2020)
LGN, *dm*Pins, *ce*GPR-1/2	HeLa, MDCK cyst/monolayer	Scaffold	Polar cortex	Misorientation Multilumen	(Mochizuki et al., 1996; Willard et al., 2004; Rodriguez-Fraticelli et al., 2010; Woodard et al., 2010; Zheng et al., 2010; Pan et al., 2013; Machicoane et al., 2014; Saadaoui et al., 2014; Carminati et al., 2016; Gloerich et al., 2017; Hart et al., 2017; Saadaoui et al., 2017; Wang et al., 2018; Pirovano et al., 2019; Takayanagi et al., 2019)
Gai, *dm*Gai/Goa, *ce*GPR-1/2	HeLa, MDCK cyst	GTPase of G-proteins	Cell cortex	Misorientation Multilumen	(Du and Macara, 2004; Chishiki et al., 2017)
Ric-8a *dm*Ric8, *ce*Ric8/synembrin	HeLa, MDCK cyst	GEF	Cell cortex, TJ	Misorientation Multilumen	(Woodard et al., 2010; Chishiki et al., 2017)
Cdc42, *dm*Cdc42, *ce*Cdc42	Caco-2/MDCK cyst	GTPase	Cell cortex, Centrosomes	Misorientation Multilumen	(Otani et al., 2006; Jaffe et al., 2008; Qin et al., 2010; Rodriguez-Fraticelli et al., 2010; Vodicska et al., 2018)
Intersectin-2	MDCK cyst	GEF	Centrosomes	Misorientation Multilumen	Rodriguez-Fraticelli et al. (2010)
Tuba	MDCK cyst	GEF	Cell cortex	Misorientation Multilumen	(Otani et al., 2006; Qin et al., 2010)
PAR1b, *dm*Par1b, *ce*PAR1	MDCK cyst, hepatocyte cells	Scaffold and adaptor	Apical cortex	Misorientation	(Lázaro-Diéguez et al., 2013; Slim et al., 2013)
PAR3, *dm*Bazooka, *ce*PAR3	Caco-2/MDCK cyst	Scaffold and adaptor	Apical cortex	Misorientation Multilumen	(Hao et al., 2010; Vorhagen and Niessen, 2014)
PAR6, *dm*PAR6, *ce*PAR6	Caco-2/MDCK cyst	Scaffold and adaptor	Apical cortex	Misorientation Multilumen	(Durgan et al., 2011; Vorhagen and Niessen, 2014)
aPKC, *dm*aPKC, *ce*PKC-3	Caco-2/MDCK cyst	Apical polarity	Apical cortex	Misorientation Multilumen	(Hao et al., 2010; Durgan et al., 2011; Vorhagen and Niessen, 2014)
SAPCD2	MDCK, Mouse retina epithelium	Apical polarity	Apical cortex	Misorientation Multilumen	Chiu et al. (2016)
Dlg1, SAP97, *dm*Dlg, *ce*DLG-1	HeLa, Caco-2/MDCK cyst, Chick neuroepithelium, Intestinal organoids Mice intestine	Polarity protein	Basolateral Cell cortex	Misorientation Multilumen	(Saadaoui et al., 2014; Porter et al., 2019; Young et al., 2019)
JAM-A	HeLa, MDCK cyst, MDCK monolayer, Murine brain	Junction formation	TJ	Misorientation Multilumen, Fate defects	(Tuncay et al., 2015; Fededa et al., 2016)
Afadin, *dm*Canoe, *ce*AFD-6	HeLa, Caco-2/MDCK cyst Hepatocyte, Mice intestine	Junction formation Actin-binding	Lateral cortex, AJ	Misorientation Multilumen, Intestine defects	(Carminati et al., 2016; Gao et al., 2017; Rakotomamonjy et al., 2017; Lough et al., 2019; Bonucci et al., 2020)
E-Cadherin, *dm*sgh, *ce*HMR-1	HeLa, MDCK cyst	AJ formation	Lateral cortex, AJ	Misorientation Multilumen	(Gloerich et al., 2017; Hart et al., 2017; Lázaro-Diéguez and Müsch, 2017; Wang et al., 2018)
IQGAP1, *ce*pes-7	MDCK cyst	Adhesion, Actin-binding, MT-binding	Basolateral Cell cortex	Misorientation Multilumen	(Bañón-Rodríguez et al., 2014; Vodicska et al., 2018)
MISP	HeLa, Caco-2 cyst	Actin and MTs interactor	Cell cortex	Misorientation Multilumen	(Zhu et al., 2013; Kschonsak and Hoffmann, 2018; Vodicska et al., 2018)
ERM, *dm*Moesin, *ce*ERM-1	HeLa, MDCK cyst	Linking Actin to cortex	Cell cortex	Misorientation Multilumen	(Hebert et al., 2012; Machicoane et al., 2014; Kschonsak and Hoffmann, 2018)
Tacc3, *dm*TACC, *ce*TAC-1	HeLa, Intestinal organoids, Murine intestine	MTs stabilization	Centrosomes, Spindle poles	Misorientation	(LeRoy et al., 2007; Burgess et al., 2015; Yao et al., 2016)

One of the first proteins to be implicated in planar divisions in cysts was the GTPase Cdc42, whose depletion in Caco-2 cells results in multi-lumen cysts due to spindle misorientation (Jaffe et al., 2008). In MDCK cells, Cdc42 has been shown to be activated by the two GEFs Tuba, regulating cell-cell junctions and Cdc42 apical localization (Qin et al., 2010; Otani et al., 2006), and Intersectin-2, implicated in endocytosis and in the mitotic Cdc42 targeting at centrosomes (Rodriguez-Fraticelli et al., 2010; Okamoto et al., 1999; Hussain et al., 2001). Planar spindle orientation is also mediated by the apically-localized polarity complex composed by Par3, Par6 and the kinase aPKC (Figure 1B). Several studies in 3D systems have shown that depletion of Par3 leads to mislocalization of the kinase aPKC (Hao et al., 2010; Zheng et al., 2010; Durgan et al., 2011; Vorhagen and Niessen, 2014), which phosphorylates LGN on Ser401 to exclude it from the apical side ensuring its localization at the lateral cortex, possibly by direct association with the baso-lateral protein Dlg-1 (Saadaoui et al., 2014). An intriguing role has been described for the Par1b/MARK2 kinase that in MDCK cells monolayer with high Rho activity promotes LGN/NuMA recruitment at the lateral site and planar divisions with the spindle axis aligned to the substratum. Conversely, in hepatocytes, that in addition to apico-basal polarity also organize a lateral lumen for the development of bile canalicular networks and have reduced Rho activity, Par1b prevents NuMA/LGN lateral recruitment causing tilted spindles and asymmetric partitioning of the lateral lumen among daughter cells (Lázaro-Diéguez et al., 2013; Slim et al., 2013).

In addition to their cohesive role, also some junctional proteins have been shown to be involved in spindle orientation in cysts, including the Junctional adhesion molecule-A (JAM-A), Afadin (AF6), E-Cadherin and Dlg-1 (Discs large homolog 1) (Figure 1B). In MDCK cysts, JAM-A activates Cdc42 and PI(3)K (Phosphatidylinositol 3-kinases), generating a gradient of PtdIns(3,4,5)P3 enriched at the cortex area facing the spindle poles, which is required for correct localization of dynein/dynactin and for spindle orientation (Toyoshima et al., 2007; Tuncay et al., 2015). Consistently, JAM-A was shown to activate Cdc42 also in progenitors of the developing cerebral cortex this way contributing to spindle orientation (Fededa et al., 2016).

The actin-binding protein Afadin, localized at adherent junctions, mediates planar spindle orientation in Caco-2 cyst by recruiting LGN to the lateral cortex via direct interactions with the LGN-TPR domain (Carminati et al., 2016; Gao et al., 2017; Bonucci et al., 2020) (Figure 1B). Consistently, studies conducted in MDCK cysts (Gao et al., 2017), hepatocyte cells (Bonucci et al., 2020), and murine neuro glia (Rakotomamonjy et al., 2017) show that Afadin is crucial for spindle orientation as its depletion leads to an aberrant spindle placement (Lough et al., 2019; Carminati et al., 2016; Gao et al., 2017; Bonucci et al., 2020; Rakotomamonjy et al., 2017). In MDCK cysts, planar cell divisions also rely on the interaction between the intra-cellular domain of E-cadherin and LGN-TPR domain (Gloerich et al., 2017; Hart et al., 2017) (Figure 1B). As with Afadin, NuMA competes also with E-cadherin for LGN binding (Zhu et al., 2011; Carminati et al., 2016; Gloerich et al., 2017; Hart et al., 2017). This suggests that Afadin and E-cadherin might be needed for the initial LGN targeting at the cortex, when NuMA is still in the nucleus, and that these interactions dissociate later in mitosis. An alternative explanation envisions that the cortical Gαi^GDP^-bound pool of LGN cycles between different mitotic binding partners associating with its TPR domain, including NuMA, Afadin and E-cadherin, in order to coordinate mechano-sensing junctional cues with spindle orientation and mitotic progression. Future live-imaging studies will clarify whether this is the case.

In addition to this role, E-cadherin was shown to be important for maintenance of cell polarity and spindle orientation in prostate epithelia by interacting with LGN, NuMA and Scrib at the lateral sites of mitotic cells, this way preserving correct apico-basal polarity, planar cell divisions and tissue integrity. Consistently, conditional loss of E-cadherin during murine prostate development leads to disorganized epithelia observed in early state prostate tumorigensis (Wang et al., 2018). Spindle orientation functions have been reported also for the baso-lateral polarity protein Dlg-1, that belongs to the membrane-associated guanylate kinase (MAGUK) family and is required for adherens junction formation and maintenance (Su et al., 2012) (Figure 1B). In HeLa cells, in MDCK cysts and in the chick neuroepithelium, Dlg-1 promotes spindle orientation by binding to the phosphorylated LGN protein (Saadaoui et al., 2014; Saadaoui et al., 2017; Porter et al., 2019), fully in line with was previously shown in *Drosophila* epithelial systems (Morin and Bellaïche, 2011; Pietro et al., 2016). In turn, the correct localization of Dlg-1 is influenced by other factors including Gαi (Saadaoui et al., 2014) and the tumor suppressor protein CASK (calcium/calmodulin-dependent serine protein kinase) (Porter et al., 2019). The binding of Dlg-1 to CASK and Gαi is key to direct LGN to restricted cortical regions before metaphase, and ultimately to target LGN and NuMA-dynein appropriately (Saadaoui et al., 2014; Saadaoui et al., 2017; Porter et al., 2019).

Another polarity protein affecting LGN cortical recruitment is the suppressor APC domain containing 2 (SAPCD2), that has been shown to interact with Gαi/LGN complexes to orchestrate mitotic spindle orientation in MDCK cyst and in mouse retina (Chiu et al., 2016). Specifically, SAPCD2 binding to the close conformation of LGN restricts LGN/NuMA accumulation at the lateral site providing a mechanism to balance the proportion of planar and vertical divisions, and hence the symmetric or asymmetric outcome of retinal progenitor mitosis (Chiu et al., 2016).

We already reported the relevance of the Gαi GEF Ric-8A for spindle orientation in HeLa cells (Woodard et al., 2010). Recent work highlighted a role for Ric-8A in tight junction formation in MDCK cysts and in LGN recruitment to the lateral cortex by generation of a localized Gαi-GDP pool promoting planar cell divisions (Chishiki et al., 2017) (Figure 1B).

Beside junctional and polarity proteins, the actin cytoskeleton, as well as actin and microtubule-binding proteins, contribute actively to spindle orientation (Pietro et al., 2016), as described in invertebrate systems such as *Drosophila* neuroblasts (Kunda and Baum, 2009) and HeLa cells (Pietro et al., 2016; Rizzelli et al., 2020). However, the role of actin in planar division and cystogenesis is less clear. In MDCK cysts, the microtubule-associated protein IQGAP1, localized at the basal site, participates to MTs dynamics and promotes planar spindle orientation by interacting with the MT plus-ends and by targeting NuMA laterally (Bañón-Rodríguez et al., 2014) (Figure 1B). Notably, in HeLa cells the interaction between IQGAP1 and Cdc42 has been shown to allow the binding of Cdc42 to the actin-binding protein MISP (Mitotic Interactor and Substrate of PLK1) implicated in spindle positioning (Zhu et al., 2013; Cadart et al., 2014; Vodicska et al., 2018). MISP associates to members of the ERM (Ezrin, Radixin and Moesin) protein family, that connects the mitotic acto-myosin cortex to the plasma membrane, in this way assisting the correct localization of NuMA at the cortex for correct spindle positioning (Hebert et al., 2012; Zhu et al., 2013; Machicoane et al., 2014; Kschonsak and Hoffmann, 2018) (Figure 1B).

### Mitotic Spindle Orientation in Intestinal Organoids

Studies of oriented divisions in cysts provided great insights into the crosstalk between orientation pathways and epithelial polarity. However, cysts of immortalized cell lines do not entirely recapitulate the cell diversity and the signaling response of epithelial tissues *in vivo* (Lancaster and Knoblich, 2014; Clevers and Tuveson, 2019).

Tissue organoids, especially murine intestinal organoids, are becoming a relevant model to study division orientation in a more physiological setting. Organoids are model systems that recapitulate not only the morphology of the organ but also the cellular composition, from stem cells to differentiated lineages (Clevers, 2013; Sato and Clevers, 2013). Methods to grow, manipulate genetically and image intestinal organoids have been first established in the Clevers lab (Sato and Clevers, 2013; Sato et al., 2009), whose work revealed that the organoids grown from intestinal epithelial cells form crypt and villi-like domains mirroring the morphology of the intestinal epithelium, with an analogous composition and distribution of cell types (Sato and Clevers, 2013; Sato et al., 2009) (Figure 1C). These studies revealed that in intestinal organoids the proliferating cells reside at the bottom of the crypt, close to the stem cell niche compartment constituted by non-dividing Paneth cells, that generate a Wnt3 gradient decreasing along the crypt axis (Figure 1C). Intestinal stem cells (ISCs) divide symmetrically moving toward the apical side of the monolayer that faces the organoid lumen, with the metaphase plate perpendicular to the apical side (Figure 1C). These ISC apical mitosis retain a connection to the basal site, and hence to the ECM, through an actin cable (Carroll et al., 2017; McKinley et al., 2018) (Figure 1C) that is essential for daughter cells to move back to the basal side of the monolayer upon cytokinesis (Carroll et al., 2017). As a matter of fact, the use of intestinal organoids to study oriented division is still in its infancy, contributed mainly by descriptive imaging experiments and a few mechanistical studies investigating the molecular mechanisms of mitosis. Little is known on molecules executing oriented divisions in organoid, but it is plausible that the same set of polarity and junctional proteins important for correct cystogenesis is implicated in division orientation also in these systems, with molecular details that remain to be explored.

Ablation of Dlg-1 from the murine intestinal crypts has been shown to result in misoriented divisions of the intestinal stem cells with a consequent delay in cell migration from the crypts bottom to the villi that promotes tumorigenic events (Young et al., 2019). Similarly, depletion from the murine crypts of the protein Tacc3, which is involved in MT crosslinking and stabilization of the Aurora-A dependent kinetochore-microtubules attachment (LeRoy et al., 2007; Burgess et al., 2015), blocks proliferation (Yao et al., 2016). Interestingly, knock-out of Tacc3 from intestinal organoids derived from APC (Adenomatous polyposis coli) mutated mice, models for colorectal cancer (Merenda et al., 2020), increases chromosome misalignment and hypomorphic mitotic spindles, leading to prolonged mitosis or mitotic arrest (Yao et al., 2016), to a certain extent mimicking what observed *in vivo*. Both findings open the possibility to target specific mitotic spindle proteins for chemotherapeutic therapy. In conclusion, although organoids hold the potential to allow more insightful analyses on the orientation pathways and their relevance for morphogenesis and disease, more studies are required to elucidate the molecular mechanisms accounting for oriented divisions in these systems.

### Spindle Misorientation: What Can Go Wrong and What Can be Done to Fix it

As discussed, oriented divisions are important for the regulation of epithelial morphogenesis and homeostasis. Consistently, their deregulation has been associated to several pathological conditions such as cancer, microcephaly, and developmental defects (Gillies and Cabernard, 2011; Nakajima, 2018; Lechler and Mapelli, 2021). However, not always the causal relationship between misorientation and diseases is clear. *In vivo* studies revealed that spindle misorientation is oftentimes corrected or is embryonic lethal (Nakajima, 2018; Lechler and Mapelli, 2021).

In murine hepatic epithelial cells *in vivo*, spindle misorientation leads to detachment of epithelial sheets from nephron epithelial tubules (Gao et al., 2017). Similarly, in stem cell systems, misorientation alters the balance between symmetric and asymmetric divisions resulting in defective changes in architecture and functioning. This has been documented for neuroepithelial progenitors during murine cortical development, in which misorientation leads to the expansion of the radial glial compartment with a delay in neurogenesis (Fededa et al., 2016).

Tissues have developed different mechanisms to rescue the damage that a misoriented spindle can cause, that have been first discovered in *Drosophila* and still await to be confirmed in mammalian tissues. The first mechanism impinges on the ability of epithelial tissue to reintegrate cells that after misoriented cytokinesis are misplaced above the epithelial layer (Bergstralh et al., 2015; Lough et al., 2019). As described for intestinal organoids (Carroll et al., 2017), in *Drosophila* imaginal disc the dividing cells have an actin protrusion that keeps them in connection to the basal side of the monolayer and assists the appropriate repositioning of daughters after cytokinesis (Nakajima et al., 2013). Parallel studies showed that also adhesive molecules, such as Fasciculin-2/3 and neuroglian, play a role in reintegrating in the epithelial layer the cells misplaced above the follicular epithelium due to orientation defects (Bergstralh et al., 2015; Cammarota et al., 2020). In *Drosophila* imaginal discs, evidence was provided that upon misorientation, one of the two daughter cells loses connection with the basal side and is displaced in the lumen (Nakajima et al., 2013). In the absence of re-integration, the misplaced cells can encounter two different fates: it either remains in the wrong position, where proliferation causes morphological defects (Dekanty et al., 2012; Nakajima et al., 2013; Poulton et al., 2014), or it undergoes apoptosis due to lack of survival signals (Nakajima et al., 2013; Poulton et al., 2014). Whether any of these mechanisms for misorientation correction is in place in vertebrate epithelial tissues remains an interesting open question.

## Conclusion

Much is known about division orientation and how the spindle orientation components are recruited to the cortex in single cells in isolation and cysts. However, a clear picture of orientation mechanisms in more complex systems, such as organoids and tissues, is still missing. The complexity of cell-cell contacts and the presence of different cell populations in epithelial tissues contribute to determine the division orientation in ways that we do not fully grasp. We also still need to further understand the mechanisms that mammalian tissues have evolved to respond to misorientation in order to preserve tissue architecture. Some of the open questions that the field should address in the future are how the correction mechanisms work in mammalian systems and how we can leverage this knowledge to better understand physio-pathological processes associated with misoriented spindles in the presence or absence of other genetic lesions. We anticipate that the use organoids as model systems might be instrumental in these studies.
